# Convention of American Anti-Tuberculosis League

**Published:** 1905-05

**Authors:** 


### CONVENTION OF AMERICAN ANTI-TUBERCULOSIS LEAGUE.

THE Anti-Tuberculosis League of America held its session in Atlanta, April 16-20. The meetings were held in the House of Delegates at the State Capitol, and were very largely attended. Nothing was omitted to contribute to the pleasure of the visiting members, and it is likely that this convention will long be remembered as one of the most elaborate and successful in the history of the League.

The association will meet next year in El Paso, Texas, the time of the convention to be announced later by the president. The annual election of officers resulted in the following manner:

President, R. E. Conniff, Sioux City, Iowa.

Secretary and Treasurer, Dr. Walter N. Vilas, of El Paso, Tex.

Vice-Presidents, Dr. Hubbard, of Georgia; Dr. Van Dyke, of

Georgia; Dr. Ambler, of North Carolina; Dr. Snodgras, of Missouri; Dr. McGhee, of Louisiana; Dr. Milikin, of Ohio; Dr. Hickey, of Michigan; Dr. Williams, of Kentucky; Dr. Porter, of Florida; Dr. Gray, of New Jersey, and Dr. McMurray, of Tennessee.

### CREED ADOPTED.

A series of resolutions, setting forth the creed of the League, was prepared by the Committee on Resolutions and formally adopted by the assembled delegates as being the sense of the convention. The resolutions follow:

"First. Tuberculosis is communicable, not inherited. A predisposition to this disease may be transmitted from parent to child, but not the disease itself.

"Second. Tuberculosis is curable. When the presence of the germ is early recognized, it may be destroyed in the individual by the adoption of proper remedial measures.

"Third. Tuberculosis is preventable. Two factors are essential to its development. First, the presence of the germ; and second, a soil favorable for its development. To some extent one or both of these factors are subject to intelligent control. The disease is, therefore, to this extent preventable.

"Fourth. We respectfully recommend the appointment by this league of three physicians in each State, whose duty it will be to form State anti-tuberculosis leagues, to invoke the aid of the profession and the State in the crusade against this dreadful disease.

"*Resolved*, That this league respectfully transmit a copy of these resolutions to the secretary of each State society, to be presented to the societies for consideration and action thereon, and asking their co-operation.

"*Resolved*, That the object sought to be accomplished by this congress of the American Anti-Tuberculosis League is to present to the American public practical and practicable methods and measures to avoid the infection of tuberculosis, to suppress its spread, and in furtherance of this end, to endeavor by education to encourage a healthful condition of living, through personal hygiene and domestic sanitation.

"*Resolved further*, That a committee be appointed by the chair to prepare concise and forcible leaflets, tracts, pamphlets and other literature intended for the information of the public, on the subject of tuberculosis, and the manner in which it is usually acquired, how to prevent its spread by proper living and proper sanitary care of the patient, and his dwelling, and for the presentation of this subject interestingly, practically, understandingly. Such literature to be distributed widely throughout the United States, in home, store, workshop and factory; be it also

"*Resolved*, That the committee so appointed shall be authorized to solicit funds by popular subscription to defray the expenses for carrying out the purpose of this resolution."

In every particular the convention was unanimously pronounced a thorough success, and Dr. George Brown, the retiring President, was freely commended for his excellent services in behalf of the association.

